# Coffee intake may promote sudomotor function activation *via* the contribution of caffeine

**DOI:** 10.3389/fnut.2022.1051828

**Published:** 2022-12-08

**Authors:** Ryeo-Won Kwon, Jin-Sun Park, Ha-Gyoung Lee, Jong-In Park, Eon-Ah Choo, Seung-Jea Lee, Jeong-Beom Lee

**Affiliations:** ^1^Department of Physiology, College of Medicine, Soonchunhyang University, Cheonan, Republic of Korea; ^2^Department of Medical Sciences, Soonchunhyang University, Asan, Republic of Korea

**Keywords:** coffee, caffeine, axon-reflex, activated sweat gland density, sudomotor function, QSART

## Abstract

**Objectives:**

To determine whether drinking coffee with caffeine accelerates the sympathetic response to acetylcholine (ACh).

**Methods:**

Tests were performed twice at 1-week intervals following the intake of coffee. Subjects were randomly divided into two groups: Group A was administered 16 fluid oz of water (CON), while Group B was given 16 fluid oz of coffee (Coffee). After 1 week, Group A was administered 16 fluid oz of coffee (Coffee), while Group B was given 16 fluid oz of water (CON). The quantitative sudomotor axon reflex test (QSART) was performed after intake of coffee and water and a 40 min break. QSART with iontophoresis and 10% ACh was performed to determine axon reflex (AXR) mediated with and without iontophoresis [AXR (1) and AXR (2), respectively], and directly activated sweating (DIR).

**Results:**

The sweat onset time of the AXR was shorter in the Coffee compared with the CON (*p* < 0.05). The sweat rates in AXR (1) AXR (2) and DIR were significantly higher in the Coffee than in the CON (*p* < 0.05, *p* < 0.05, *p* < 0.01, respectively). In addition, the Coffee showed significantly higher density of activated sweat glands and activated sweat gland output than the CON (*p* < 0.05, *p* < 0.01, respectively). The overall results of this study showed that coffee intake could stimulate higher activation in both AXR and DIR sweat responses.

**Conclusion:**

Coffee intake can improve sweating sensitivity in both the AXR and DIR by the contribution of caffeine contained in coffee. This suggests that other compounds in coffee may not inhibit the sympathetic response to ACh. Therefore, coffee may be clinically worth considering as a supplement for the activation of the cholinergic and sudomotor function.

## Introduction

Modern society consumes a lot of coffee during business hours and in leisure time (1). Coffee intake is common in everyday life, and the demand is increasing all the time. Coffee contains large or small amounts of caffeine. Caffeine is a psychoactive agent that is used worldwide because it exists in various foods, including tea, drinks, chocolate, and coffee (2). Caffeine increases concentration and improves performance, and in particular, it is effective in improving cognitive function and overcoming decline in physical performance induced by lack of sleep (3). In addition, coffee and/or caffeine intake has been associated with a reduced risk of cardiovascular diseases (4, 5), and various cancers such as melanoma (6), breast cancer (7), non-melanoma skin cancer (8). Additionally, coffee and/or caffeine intake may prevent obesity by increasing thermogenesis and energy expenditure (9, 10).

Caffeine is an antagonist of the adenosine A1 and A2A receptors (1), and it can increases cholinergic activation (11, 12). In our previous study, 3 mg ⋅ kg^–1^ caffeine intake stimulated higher sweating-related thermogenesis even with the same physical activity, resulting in significantly higher local activated sweat gland density (ASGD), whole-body sweat loss volume, and lipolysis (11). A subsequent study also found that physical exercise with 3 mg ⋅ kg^–1^ caffeine intake significantly increased activated sweat gland output (ASGO) locally including chest, abdomen, thigh, and back (12). In addition, the sweat onset time was significantly faster, indicating that caffeine intake accelerated the sympathetic response to acetylcholine (ACh) (12).

Sweating is a physiological activity essential for maintaining homeostasis of body temperature, which may be elevated by excessive exercise and heat exposure. Further, sweating may occur in response to emotional stress and result in the expulsion of waste products as well as maintain skin moisture to control skin homeostasis (13). Approximately 4 million sweat glands are distributed in the human integument, and the development of sweat glands is completed within 2.5 years after birth (14). Sweat secretion, is regulated by transmitting signals from the central nervous system (CNS) to the peripheral nervous system (PNS), particularly the autonomic nervous system (ANS) (13).

The clinical evaluation of sudomotor function has become a standard component of clinical autonomic functional tests evaluating the integrity of the cholinergic segment of the sympathetic nervous system (SNS) (15). Sudomotor function can be evaluated *via* thermoregulatory sweat testing, quantitative sudomotor axon reflex sweat test (QSART), silicon imprint, and electrochemical skin conductance. QSART can analyze the indirect axon reflex (AXR) *via* direct activation (DIR) of muscarinic receptors in sweat glands with cholinergic stimulation and nicotine receptors at the nerve endings in sweat glands (16–18).

Caffeine intake enhances sweating sensitivity *via* cholinergic activation by QSART (12). However, the previous study did not confirm the sweat response that differentiated AXR and DIR (12). In addition to caffeine, coffee also contains polyphenols such as chlorogenic acids and lignans, cafestol, kahweol, trigonelline, melanoidins, and traces of magnesium, potassium, and vitamins B3 (19). Since no study to date has investigated the effects of coffee intake on the sweating response of AXR and DIR, the current study was practiced. Therefore, the purpose of this study was to determine whether the sympathetic nerve response to ACh is accelerated by the intake of coffee containing a large amount of caffeine. The sweat gland count, rate and function following coffee intake could be used for clinical diagnosis of motor function in healthy individuals.

## Materials and methods

### Subjects

The study included 40 healthy, male non-athletic volunteers (age, 22.38 ± 3.18 years; height, 174.4 ± 4.69 cm; weight, 69.72 ± 5.08 kg). Inclusion criteria were: (1) absence of major health problems, such as diabetes, heart diseases, metabolic disorders; (2) absence of diseases affecting skin and neuropathy and medication use that may directly influence the experimental results; (3) lack of habitual caffeine intake (Self-reported average daily caffeine [include chocolate, tea, energy drink, coffee, etc.] intake between 0 and 250 mg for the past 3 months); (4) absence of side effects due to caffeine; and (5) no smoking history. All subjects were asked to refrain from coffee intake (include chocolate, tea, energy drink, coffee, etc.) smoking, alcohol, medication use, exercise, and excessive heat exposure 48 h before the test. Each subject provided written informed consent to participate in this study, following explanation of the purpose, experimental procedures, and any potential risks. All subjects read and signed informed consent regarding the study purpose and procedure. This work was supported and supervised by the Soonchunhyang University Research Fund and complies with the tenets of 2013 Declaration of Helsinki of the World Medical Association and the experimental protocol recommended by the University of Soonchunhyang Research Committee. Studies involving human participants were reviewed and approved by the Institutional Review Board on Human Subjects Research and Ethics Committees, Soonchunhyang University (Nos. 1040875-201611-BR-042 and 1040875-202104-BR-030).

### Measurements and experimental procedures

The tests were performed in a climate chamber at 25.0 ± 0.5^°^C, 60.0 ± 3.0% relative humidity, and 1 m/s air velocity. The subjects sat in a chair in a relaxed posture for 20 min in order to acclimatize to the chamber prior to the commencement of the experiments.

**SCHEME 1:**
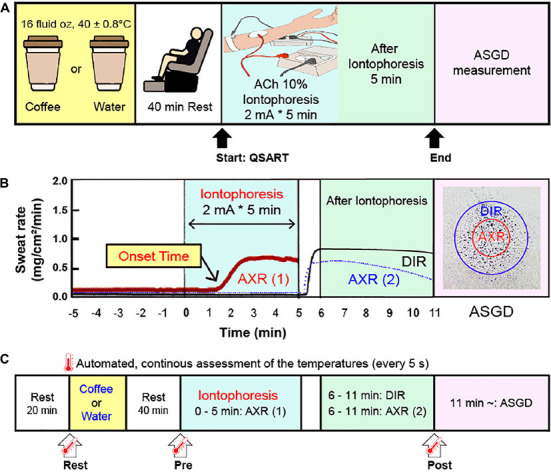
**(A)** Schematic representation of the experimental protocol. Subjects consumed coffee or water 40 min before the start of the quantitative sudomotor axon reflex test (QSART). After QSART was ended, activated sweat gland density (ASGD) was measured. **(B)** Schematic from a single subject data sample showing axon reflex (AXR) (1) (shown with the red solid line), AXR (2) (shown with the blue dotted line) and direct activation responses (DIR, shown with the black solid line) by QSART. **(C)** Schematic of the time point of body temperature. Tympanic and local skin temperature of the chest, upper arm, thigh, and leg measurements were conducted continuously at 5 s intervals during the experiment (from immediately before intake coffee or water to 6 min after QSART). The temperature changes of the two groups were compared at three points: Rest, immediately before coffee or water intake; Pre, 40 min after coffee or water intake (immediately before QSART); Post, 6 min after QSART (immediately after all DIR and AXR (1), AXR (2) measurements have been completed). QSART, quantitative sudomotor axon reflex test; ACh, acetylcholine; AXR, axon reflex mediated (indirectly activated) sweating during (nicotinic receptor mediated sweating activity); AXR (1), AXR sweating during 5 min 10% ACh iontophoresis; AXR (2), AXR sweating during 5 min post-iontophoresis; DIR, directly activated sweating during (muscarinic receptor mediated sweating activity) 5 min post-iontophoresis.

The test used a random, crossover design. Tests were performed twice at 1 week intervals following the intake of coffee. We pursued the experiment between 2 and 5 p.m to restrict for the affection of circadian rhythm on body temperature (20). Subjects were randomly divided into two groups: Group A was administered 16 fluid oz of water (CON), while Group B was given 16 fluid oz of coffee (Coffee). All subjects sat in a chair in a relaxed posture for 40 min and QSART was initiated. After 1 week, Group A was administered Coffee, while Group B was given CON. All subjects were tested again similarly. The experimental protocol is presented in Scheme 1A.

Coffee and water were provided to the subjects, in the same volume and temperature, in an invisibly packaged opaque shaker bottle of the same shape, without guiding the contents. Participants were instructed to drink coffee or water within 5 min.

#### Coffee dose

In humans, 99% of caffeine is absorbed within 45 min, and plasma caffeine levels peak about 40–60 min after intake (21, 22). Therefore, we performed QSART 40 min after intake of coffee. The coffee used in this study was caffe americano (Starbucks, Seoul, Republic of Korea) Grande size (16 fluid oz, 473 mL) containing 225 mg caffeine. The product nutritional information is: caffeine 225 mg, carbohydrate 3 g, sodium 10 mg, protein 1 g, 15 kcal. According to the US Dietary Guidelines Advisory Committee and European Food Safety Authority, the maximum recommended daily intake of caffeine for adults is 400 mg, with 3–5 cups of coffee per day reporting no health effects (23, 24). The temperature of coffee and water provided to the participants was 40 ± 0.8^°^C to minimize the effect of blood flow temperature in vascular (25).

#### Tympanic temperature and mean body temperature

The Tympanic temperature (TYMP) and mean body temperature (mT_*b*_) were performed in a similar method as used in previous studies (12, 20, 26, 27). To calculate the TYMP, mT_*b*_, and mean skin temperature (mT_*s*_) were measured (12, 20, 26, 27). The mT_*b*_ was calculated using the formula of Sugenoya and Ogawa (28): mT_*b*_ = (0.9 ⋅ TYMP + 0.1 ⋅ mT_*s*_); and mT_*s*_ was calculated using the Ramanathan equation (29). TYMP, skin temperature of the chest, upper arm, thigh, and leg were measured continuously at 5 s intervals during the course of the experiment (from immediately before intake coffee or water to 6 min after QSART) in the left ear by inserting a thermistor probe (TSK7 + 1, Songkitopia, Incheon, Republic of Korea) with a small spring (K923, Takara, Yokohama, Japan) interlocked to a personal computer (CF-T1, Panasonic, Tokyo, Japan) and a data logger (K-720, Technol Seven, Yokohama, Japan). When the contacting tympanic membrane with the thermistor probe may cause discomfort and scratching sound hear to the subject (12, 20, 26, 27). The inner pinna was filled with small cotton balls to fix the probe in the ear (12, 20, 26, 27). Comparisons of TYMP and mT_*b*_ changes in the two groups were made at three points (Scheme 1C): Rest, immediately before coffee or water intake; Pre, 40 min after coffee or water intake (immediately before QSART); Post, 6 min after QSART (immediately after all DIR and AXR (1), AXR (2) measurements have been completed).

#### Quantitative sudomotor axon reflex test

QSART was performed in the similar method as used in previous studies (17, 30–32). For quantification of sweat secretion by QSART, 10% ACh chloride solution was used as a neurotransmitter. The QSART capsule consists of three concentric compartments (17, 30–32). Iontophoretically applied ACh stimulates the underlying sweat glands in the outer compartment directly while the glands of the skin in the central compartment of the capsule are activated indirectly *via* AXR (17, 30–32). The sweating response was measured from the directly activated and AXR-mediated sweat responses resulting from the iontophoresis (17, 30–32). Two sets of QSART capsules were attached to the volar aspect of the forearm with rubber bands, one at the mid portion between the wrist and elbow joints and the other 10 cm proximal to the first (17, 30–32). The outer compartment of the first capsule was filled with 10% ACh solution (17, 30–32). Direct current (2 mA) was applied for 5 min between an electrode on the ACh cell (anode) and a flexible plate-electrode (cathode) attached to the forearm skin just proximal to the wrist joint (17, 30–32). The sudomotor AXR (1) was assessed during iontophoresis *via* central section of the ACh capsule (17, 30–32). Immediately after the cessation of current loading, the sweat capsules were detached and the skin that had been covered with the ACh capsule was wiped; the positions of the capsules were then exchanged (17, 30–32). This procedure took less than 20 s (17, 30–32). Data was acquired for a second 5 min period to permit the simultaneous observation of DIR and AXR (2) sweating (17, 30–32). The data including sweat onset-time, latent period for sweating after current loading, sweat volume for 5 min, 0–5 min for AXR (1), and 6–11 min for AXR (2) and DIR were used for the analysis (Scheme 1B) (17, 30–32). Sweat onset time was measured conforming to the capacitance hygrometer-ventilated capsule method (17, 30–32). Sweat rate was continuously recorded on equivalent method. Sweat rate was recorded with a PC (model CF-T1, Panasonic, Tokyo, Japan) at a time every 5 s (17, 30–32). To summarize the capacitance hygrometer-ventilated capsule method, nitrogen gas was flowing into each compartment at a steady flow rate of 300 ml/min (17, 30–32). Measure the change in relative humidity of the effluent gas with a hygrometer (H211, Technol Seven, Yokohama, Japan) (17, 30–32). Iodine-starch paper was used for ASGD measurement (33). Iodine-starch paper was placed on the skin surface where the ACh was applied (17, 30–33). The number of blue-black pigmented spots in 0.5 × 0.5 cm areas were counted under a microscope in triplicate, and the average sweat gland density (count ⋅ cm^–2^) was calculated (17, 30–33). The sweat output per single gland (μg ⋅ min^–1^ ⋅ gland^–1^) was acquired by dividing the sweat rate (mg ⋅ cm^–2^ ⋅ min^–1^) by the sweat gland density (17, 30–33). Skin temperatures just beside and 10 can away from the ACh capsule were monitored using thermistors (TSK7 + 1, Songkitopia, Incheon, Republic of Korea) connected to data logger (K-720, Technol Seven, Yokohama, Japan) (17, 30–32). Table 1 shows comparison of skin temperature 10 cm distal and ACh capsule between the CON and Coffee groups in the QSART.

**TABLE 1:** Comparison of skin temperature 10 cm distal and acetylcholine (ACh) capsule between the CON and Coffee groups in the quantitative sudomotor axon reflex test (QSART).

		Pre	Post
10 cm distal (^°^C)	CON	33.26 ± 1.06	33.28 ± 1.17
	Coffee	33.29 ± 1.09	33.32 ± 1.25
ACh capsule (^°^C)	CON	33.21 ± 1.20	33.59 ± 1.29^+++^
	Coffee	33.23 ± 1.16	33.63 ± 1.34^+++^

The values are the mean ± SD. Pre (start-QSART), Post (5 min after beginning QSART). ^+++^*P* < 0.01, indicates a significant different from rest in the each conditions. QSART, quantitative sudomotor axon reflex test.

### Statistical analysis

Measurements were expressed as the mean ± standard deviation. For the comparison between groups, the independent sample *t*-test was used. The level of significance was set at *p* < 0.05.

## Results

### Tympanic temperature and mean body temperature

Tympanic temperature and mT_*b*_ were compared at Rest, Pre-QSART, and Post-QSART time points. TYMP and mT_*b*_ of Coffee group were higher in Pre and Post compared to Rest, but there was no significant difference between Pre and Post. There was no significant difference in TYMP and mT_*b*_ in the CON group at Rest, Pre, and Post.

### Onset time, nicotinic indirect axon reflex sweating in quantitative sudomotor axon reflex test

**FIGURE 1:**
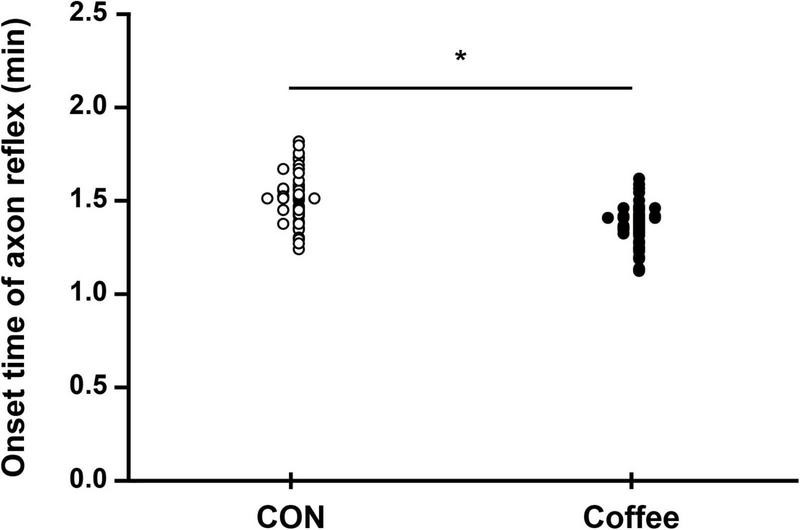
Comparison of sweating activity between CON and Coffee groups showing the sweat onset-time of axon reflex (AXR) [threshold or latency of sweating AXR (1)]. Statistical significance difference, *p < 0.05. CON, no coffee group (*n* = 40); Coffee, coffee intake group (*n* = 40).

The sweat onset time of the AXR (nicotinic receptor mediated) was significantly shorter in the Coffee group (1.38 ± 0.10 min) compared with the CON group (1.52 ± 0.16 min) (*p* < 0.05, Figure 1). The sweat onset time of quintile in the Con group was Min, 1.26; 1st quintile (Q1), 1.40; 2nd quintile (Q2), 1.50; 3rd quintile (Q3), 1.55; 4th quintile (Q4), 1.65; Max, 1.81. The sweat onset time of quintile in the Coffee group was Min, 1.14; Q1, 1.29; Q2, 1.36; Q3, 1.41; Q4, 1.46; Max, 1.61.

### Sweat rate, nicotinic indirect axon reflex and muscarinic direct sweating in quantitative sudomotor axon reflex test

**FIGURE 2:**
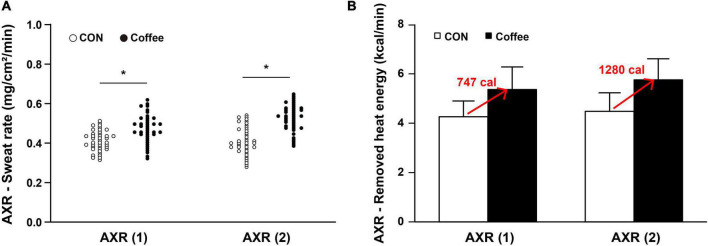
**(A)** Comparison of sweating activity between subjects in CON and Coffee groups at axon reflex (AXR) (1) and AXR (2). **(B)** Comparison of estimated value on remove heat energy of whole body between CON and Coffee groups at AXR (1) and AXR (2). AXR (1) = 0–5 min and AXR (2) = 6–11 min. Values are means + SD. Statistical significance difference, *p < 0.05. CON, no coffee group (*n* = 40); Coffee, coffee intake group (*n* = 40); AXR, axon reflex mediated; AXR (1), AXR sweating during 5 min 10% acetylcholine (ACh) iontophoresis; AXR (2), AXR sweating during 5 min post-iontophoresis.

**FIGURE 3:**
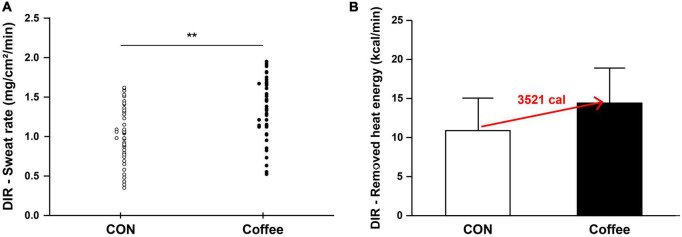
**(A)** Comparison of sweating activity of subjects in CON and Coffee groups showing direct activation (DIR) of sweat response. **(B)** Comparison of estimated value on remove heat energy of whole body between CON and Coffee groups. Values are means + SD. Statistical significance difference, **p < 0.01. CON, no coffee group (*n* = 40); Coffee, coffee intake group (*n* = 40); DIR, directly activated sweating.

The sweat rates of AXR (1), AXR (2), and DIR were all significantly higher in the Coffee group than in the CON group. During the nicotinic receptor-mediated sweating activity (indirect AXR, AXR) (1) (measurement with iontophoresis, 0–5 min for AXR), the sweat rate was higher in the Coffee group (0.47 ± 0.08 mg ⋅ cm^–2^ ⋅ min^–1^) than in the CON group (0.40 ± 0.06 mg ⋅ cm^–2^ ⋅ min^–1^) (*p* < 0.05, Figure 2A). In addition, the AXR (2) (measurement without iontophoresis, 6–11 min for AXR) sweat rate was higher in the Coffee group (0.54 ± 0.08 mg ⋅ cm^–2^ ⋅ min^–1^) than in the CON group (0.42 ± 0.07 mg ⋅ cm^–2^ ⋅ min^–1^) (*p* < 0.05, Figure 2A). During the muscarinic receptor-mediated sweating activity (direct response, DIR), the sweat rate was higher in the Coffee group (1.35 ± 0.42 mg ⋅ cm^–2^ ⋅ min^–1^) than in the CON group (1.02 ± 0.39 mg ⋅ cm^–2^ ⋅ min^–1^) (*p* < 0.01, Figure 3A).

The sweat rate AXR (1) of quintile in the Con group was Min, 0.31; Q1, 0.36; Q2, 0.38; Q3, 0.41; Q4, 0.44; Max, 0.49. The sweat rate AXR (1) of quintile in the Coffee group was Min, 0.32; Q1, 0.41; Q2, 0.45; Q3, 0.49; Q4, 0.52; Max, 0.61. The sweat rate AXR (2) of quintile in the Con group was Min, 0.29; Q1, 0.37; Q2, 0.40; Q3, 0.42; Q4, 0.50; Max, 0.55. The sweat rate AXR (2) of quintile in the Coffee group was Min, 0.39; Q1, 0.48; Q2, 0.52; Q3, 0.55; Q4, 0.58; Max, 0.65. The sweat rate DIR of quintile in the Con group was Min, 0.35; Q1, 0.73; Q2, 0.95; Q3, 1.01; Q4, 1.37; Max, 1.62. The sweat rate DIR of quintile in the Coffee group was Min, 0.52; Q1, 1.03; Q2, 1.25; Q3, 1.46; Q4, 1.68; Max, 1.95.

### Active sweating gland density and single sweat gland output

**FIGURE 4:**
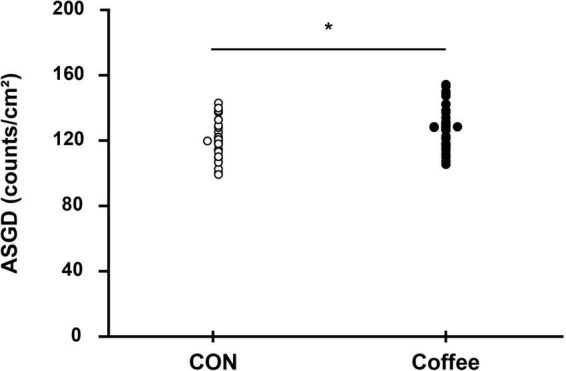
Comparison of sweating activity gland density between subjects in the CON and Coffee groups. Statistical significance difference, *p < 0.05. CON, no coffee group (*n* = 40); Coffee, coffee intake group (*n* = 40); ASGD, activated sweat gland density.

**FIGURE 5:**
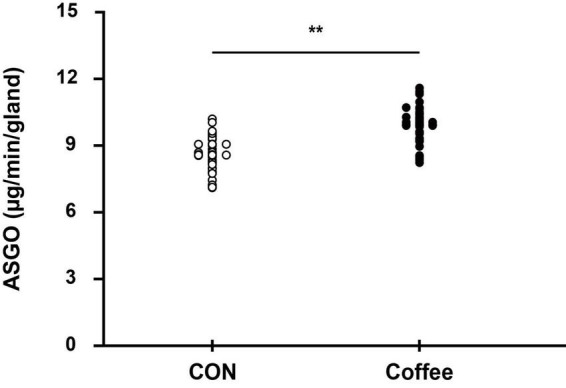
Comparison of sweating between CON and Coffee subjects showing an active sweat output per gland. Statistical significance difference, **p < 0.01. CON, no coffee group (*n* = 40); Coffee, coffee intake group (*n* = 40); ASGO, activated sweat gland output.

The active sweat gland density was higher in the Coffee group (131.52 ± 13.46 count ⋅ cm^–2^) than in the CON group (119.36 ± 12.82 count ⋅ cm^–2^) (*p* < 0.05, Figure 4). The sweat output per gland was higher in the Coffee group (10.26 ± 0.73 μg ⋅ min^–1^ ⋅ gland^–1^) than in the CON group (8.54 ± 0.65 μg ⋅ min^–1^ ⋅ gland^–1^) (*p* < 0.01, Figure 5).

The active sweat gland density of quintile in the Con group was Min, 94.99; Q1, 110.27; Q2, 117.35; Q3, 119.39; Q4, 128.28; Max, 145.23. The active sweat gland density of quintile in the Coffee group was Min, 105.98; Q1, 119.53; Q2, 130.01; Q3, 131.49; Q4, 140.28; Max, 158.45. The sweat output per gland of quintile in the Con group was Min, 7.21; Q1, 8.00; Q2, 8.52; Q3, 8.63; Q4, 8.98; Max, 10.01. The sweat output per gland of quintile in the Coffee group was Min, 8.65; Q1, 9.68; Q2, 10.23; Q3, 10.36; Q4, 10.74; Max, 11.81.

## Discussion

Since Coffee, which is high in caffeine, also contains a variety of other compounds, it is still debatable whether it can generate the full efficacy of caffeine. Caffeine controls signal transduction *via* receptors in various ways, for instance, as an antagonist of adenosine receptors (34). The adenosine antagonist effect is inhibited by chlorogenic acid, another component of coffee (35). Other physiological effects of caffeine may also antagonize the action of coffee due to the presence of various compounds (36). Graham et al. (36) reported no caffeine-like ergogenic effects after coffee intake, whereas another study reported significant ergogenic effects after coffee intake (37, 38).

Ingestion of 3 mg ⋅ kg^–1^ caffeine may increase sweating response *via* QSART (11). Therefore, in this study, we determined the effect of coffee intake with caffeine content (3 mg ⋅ kg**^–^**^1^) similar to our previous study evaluating sweating response *via* QSART. Our results show increased activation of SNS through the stimulation of ACh after coffee intake as well as caffeine intake. That is, we confirmed that coffee intake may improve ACh sensitivity separately or together with body temperature rise.

Consumption of coffee and caffeine increases body temperature and skin temperature (39, 40). In our data, the Coffee group exhibited higher body temperature in Pre, before QSART was measured, compared to the CON group (data not shown). The difference in body temperature between the two groups in Pre, before performing iontophoresis, is inevitable. Therefore, we conducted QSART after the coffee group consumed coffee and the body temperature increased enough. There was no significant change in body temperature in either group between Pre and Post, during which iontophoresis was applied and data were collected. In addition, there was no significant difference between the two groups by ACh in the local skin temperature to which iontophoresis was applied in the Coffee group and the CON group. This means that there was no significant body temperature effect during our measurements for at least 11 min with iontophoresis applied between the Coffee group and the CON group. Importantly, QSART is a method for testing autonomic function independent of body temperature.

**SCHEME 2:**
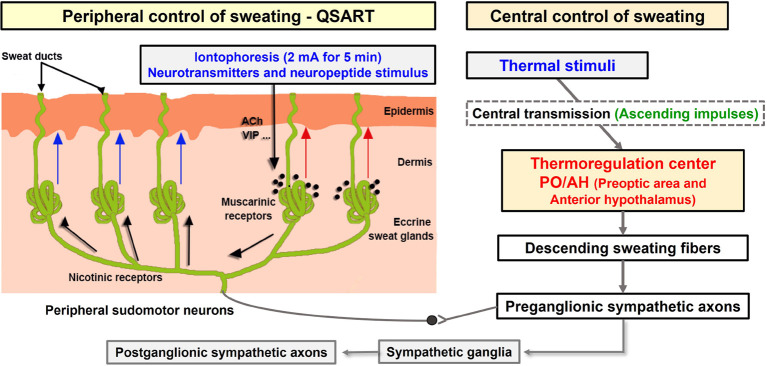
Schematic of the sudomotor axon reflex *via* iontophoresis. Cholinergic agonists (such as acetylcholine) and neuropeptides (such as vasoactive intestinal polypeptide) administered *via* iontophoresis (shown with the black arrow) bind to muscarinic receptors causing local sweat production (direct sweat, red arrow). The cholinergic agonist simultaneously binds to nicotinic receptors on the nerve terminals of sudomotor fibers triggering an antidromic impulse. This impulse travels orthodromically to a neighboring population of eccrine sweat glands at branch points resulting in an indirect axon-mediated sweat response (indirect sweat, blue arrows) (18). Modified model by Kwon et al. ACh, acetylcholine; VIP, vasoactive intestinal polypeptide. QSART, quantitative sudomotor axon reflex test.

The QSART induces axon-reflex *via* iontophoresis of ACh and used to evaluate the sudomotor function (15). ACh is a major neurotransmitter that stimulates sweat secretion. ACh has vasodilatory, axon-reflex, and eccrine sweat-stimulating functions in vascular, sensory and sympathetic C-fibers, eccrine sweat glands, anti-inflammation, respectively *via* cholinergic pathway (41–44). ACh and its derivatives activate nicotinergic and muscarinergic ACh receptors with or without changes in body temperature, leading to activation of cholinergic functions such as sweating (42, 45, 46). Therefore, QSART can be used to analyze AXR *via* DIR of muscarinic receptors in sweat glands following stimulation of cholinergic and nicotine receptors at the nerve endings in sweat glands (16). AXR involves the activation of surrounding sweat glands receiving antidromic signals *via* other branches of the same axis (18) (Scheme 2). To confirm this, we measured and compared the onset-time at AXR (1), in CON and Coffee group, respectively.

The sweat onset time of the AXR (nicotinic receptor mediated) was shorter in the Coffee group compared with the CON group (Figure 1). These results are partially consistent with the study that found a significantly shorter sweating onset time after QSART in the caffeine-ingested group than in the no-caffeine group (12). However, a previous study reported only the effect of caffeine intake, not coffee, on sweating response, regardless of DIR, and AXR. Sweating is induced by stimulation of the CNS and regulated by signals transmitted to the PNS and body temperature through release from SNS cholinergic fibers, and activation of the eccrine glands (13). Large amounts of 1, 3, 7-trimethylxanthine, the most widely used CNS stimulant, were detected in caffeine. Caffeine contained in coffee can lower the sweating of threshold by activating stimulating the CNS (12). In addition, caffeine can activate SNS stimulation that can reduce the sweat of threshold (11). This may be the contribution of caffeine-induced stimuli to the CNS and SNS. Our results show that coffee intake shortened the onset of sweating in the AXR (Figure 1), along with a reduction in threshold sweating. In addition, this suggests that coffee intake may activate nicotinergic ACh receptors.

Consistently, the coffee group showed significantly higher ASGD compared with the CON group (Figure 4). Reduction of the sweating threshold by coffee intake onsets sweating more quickly, which can activate more sweat glands at the same period section. Likewise, in our other study, during the same physical exercise after the intake of 3 mg ⋅ kg^–1^ caffeine, a significantly higher ASGD was observed in chest, abdomen, thigh, and back (12). This suggests that intake of caffeinated coffee may affect sudomotor function of the whole body as well as the forearm. Interestingly, the coffee group showed significantly higher ASGO compared with the CON group (Figure 5). Results for ASGO per gland showed that the activation of sudomotor function by coffee intake not only lowered the sweating threshold but also persisted thereafter. Activation of the sweat response initially increases ASGD and then increases the sweat rate by increasing ASGO per sweat gland. Although it is not possible to say for sure how long the effect of caffeine lasted, it is suggested that it may persist for less than 11 min or longer after QSART.

We measured the sweat rates of AXR and DIR to investigate the effect of coffee more accurately on sudomotor function, respectively. The sweat rates of AXR (1), AXR (2), and DIR were all significantly higher in the Coffee group than in the CON group (Figures 2A, 3A). These results suggest that the large amount of caffeine in coffee increased the cholinergic activation. Adenosine receptors A (1) and A (2A) regulated the release of ACh in mice (47). Similarly, treatment with caffeine, an adenosine antagonist, increased ACh dialysate concentrations in rat brain (48). Chlorogenic acids in coffee may antagonize the adenosine antagonist effects of caffeine (35), which is contrary to our findings. The coffee group appear to may improve motor function *via* cholinergic activation under the contribution of caffeine.

The sweat rate is the product of ASGD and the amount of secretion per sweat gland, and the whole body sweat rate can be assumed using the body surface area (BSA) (49). In many previous studies that measured QSART in the forearm, it was applied to estimate the sweating activity of the whole body and was used to explain sudomotor function and sensitivity of the whole body (50, 51). Completely vaporized sweat per 1 kg is equivalent to 580 kcal of heat energy being removed from the body (52). Whole body sweating was estimated by using the subject’s average BSA for the sweat rate data of AXR and DIR. Then, the estimated value on remove heat energy of whole body was calculated (Figures 2B, 3B). △Count calculate = Coffee–CON. ΔCount [AXR (1) 0.07 mg; AXR (2) 0.12 mg; DIR 0.33 mg] (*BSA *0.58 kcal/ml) = Coffee makes a contribution to evaporation, removing a total heat energy of [AXR (1) 747.04 cal; AXR (2) 1280.64 cal; DIR 3521.76 cal] per min (Figures 2B, 3B). This suggests that coffee intake elicited a higher sweat response even with the same during 5 min 10% ACh iontophoresis, which could lead to higher energy metabolism, both in AXR (Figure 2B) and DIR (Figure 3B). Caffeine intake has been shown to increase thermogenesis and increase energy expenditure in human and animal models. Thus, intake of caffeine and/or coffee not only increased thermogenesis but also increased energy consumption and metabolic rate (39, 40, 53). In a recent study, caffeine-treated rats subjected to treadmill walking showed a significant increase in activity-related energy expenditure compared with control rats (54). In addition, caffeine intake only at the end of the treadmill test led to a significant dissipation in muscle heat compared with control rats, concluding that caffeine increased the caloric cost of the physical activity (54). *In vivo* and *in vitro* studies of caffeine-induced brown adipose tissue (BAT) showed that stem cell-derived adipocytes exposed to caffeine induce BAT *via* uncoupling protein abundantly and enhanced cell metabolism with increased oxygen consumption (10). The study also confirmed the thermogenic effect on the supraclavicular region, a major area of BAT, following coffee intake by adult humans (10).

Although the effect of an increase in body temperature cannot be completely excluded, our data show that coffee intake may be sufficient to improve sudomotor function through the contribution of caffeine. In addition, other compounds in coffee may not inhibit the sympathetic response to ACh. Therefore, coffee may be clinically worth considering as a supplement for activation of sudomotor function. Interestingly, the overall results of our study showed that coffee intake could stimulate higher activation in both AXR (1), AXR (2), and DIR sweat responses. This suggests that coffee intake may activate nicotinergic [AXR (1), AXR (2), Figure 2A] and muscarinergic (DIR, Figure 3A) ACh receptors. Although further studies are needed, it is expected that coffee intake may lead to beneficial effects of cholinergic functions, such as anti-inflammatory effects of ACh in macrophage regulation and skin function (42–44). Therefore, studies on the relationship between coffee intake and cholinergic and sudomotor functions will be clinically useful. However, our study has some limitations. Because our study design did not include a group consuming decaffeinated coffee, it cannot be concluded that caffeine alone affected the enhancement of sudomotor function by coffee intake. It is not clear from our study whether other compounds in coffee promote or interfere with these effects of caffeine. In addition, the various types of coffee depending on the degree of roasting and additives such as syrup and cream can have clinical implications.

## Data availability statement

The original contributions presented in this study are included in the article/supplementary material, further inquiries can be directed to the corresponding author.

## Ethics statement

The studies involving human participants were reviewed and approved by the Institutional Review Board on Human Subjects Research and Ethics Committees, Soonchunhyang University. The patients/participants provided their written informed consent to participate in this study.

## Author contributions

R-WK wrote the manuscript with the rest of contributing revisions and input from J-SP, H-GL, J-IP, E-AC, S-JL, and J-BL. All authors contributed to scientific review and discussion of the manuscript.
